# Free-running cardiac magnetic resonance fingerprinting: Joint T1/T2 map and Cine imaging

**DOI:** 10.1016/j.mri.2020.02.005

**Published:** 2020-05

**Authors:** O. Jaubert, G. Cruz, A. Bustin, T. Schneider, P. Koken, M. Doneva, D. Rueckert, R.M. Botnar, C. Prieto

**Affiliations:** a School of Biomedical Engineering and Imaging Sciences, King's College London, London, United Kingdom; b Philips Healthcare, Guilford, United Kingdom; c Philips Research Europe, Hamburg, Germany; d Department of Computing, Imperial College London, London, United Kingdom; e Escuela de Ingeniería, Pontificia Universidad Católica de Chile, Santiago, Chile

**Keywords:** Cardiac MRI, MR fingerprinting, T1 mapping, T2 mapping, Dynamic cine imaging

## Abstract

**Purpose:**

To develop and evaluate a novel non-ECG triggered 2D magnetic resonance fingerprinting (MRF) sequence allowing for simultaneous myocardial T_1_ and T_2_ mapping and cardiac Cine imaging.

**Methods:**

Cardiac MRF (cMRF) has been recently proposed to provide joint T_1_/T_2_ myocardial mapping by triggering the acquisition to mid-diastole and relying on a subject-dependent dictionary of MR signal evolutions to generate the maps. In this work, we propose a novel “free-running” (non-ECG triggered) cMRF framework for simultaneous myocardial T_1_ and T_2_ mapping and cardiac Cine imaging in a single scan. Free-running cMRF is based on a transient state bSSFP acquisition with tiny golden angle radial readouts, varying flip angle and multiple adiabatic inversion pulses. The acquired data is retrospectively gated into several cardiac phases, which are reconstructed with an approach that combines parallel imaging, low rank modelling and patch-based high-order tensor regularization. Free-running cMRF was evaluated in a standardized phantom and ten healthy subjects. Comparison with reference spin-echo, MOLLI, SASHA, T_2_-GRASE and Cine was performed.

**Results:**

T_1_ and T_2_ values obtained with the proposed approach were in good agreement with reference phantom values (ICC(A,1) > 0.99). Reported values for myocardium septum T_1_ were 1043 ± 48 ms, 1150 ± 100 ms and 1160 ± 79 ms for MOLLI, SASHA and free-running cMRF respectively and for T_2_ of 51.7 ± 4.1 ms and 44.6 ± 4.1 ms for T_2_-GRASE and free-running cMRF respectively. Good agreement was observed between free-running cMRF and conventional Cine 2D ejection fraction (bias = −0.83%).

**Conclusion:**

The proposed free-running cardiac MRF approach allows for simultaneous assessment of myocardial T_1_ and T_2_ and Cine imaging in a single scan.

## 1 Introduction

Cardiac Magnetic Resonance (CMR) imaging has become a key non-invasive diagnostic tool to assess anatomical structures, characterize myocardial tissue and evaluate functional and mechanical properties of the heart [1]. CMR is the current gold standard for the assessment of left ventricular function and viability, based on Cine and late-gadolinium enhancement imaging, respectively. A large range of cardiac diseases also involve myocardial fibrosis, inflammation and oedema. Recently quantitative mapping of tissue parameters, such as T_1_ and T_2_ relaxation times, have been introduced to non-invasively characterize these biological processes in the myocardial tissue, promising to enable early risk assessment and therapy monitoring [[2], [3], [4]].

Conventional quantitative MR relaxometry techniques usually acquire multiple good quality images at different points along the T_1_ and T_2_ relaxation process. A parametric map is then obtained by fitting the reconstructed images to the corresponding exponential relaxation model on a pixel-wise basis. However, these techniques require longer acquisition times than conventional qualitative (e.g. T_1_-weighted, T_2_-weighted) MR imaging. Multiple parametric maps, usually required to investigate the variety of clinical manifestations of the disease, are acquired sequentially, further increasing the scan time. Moreover, these measurements are susceptible to system imperfections and other confounding factors (e.g. inter-parameter dependency).

Recently, simultaneous estimation of T_1_ and T_2_ parameters has been proposed to accelerate myocardial tissue characterisation and correct for inter-parameter induced biases in the measurements. A balanced steady state free precession (bSSFP) Look-Locker acquisition (CABIRIA) [5] with joint T_1_ and T_2_ fitting has been recently proposed, showing good agreement to MOLLI [6] and T_2_ prepared bSSFP [7] mapping in terms of accuracy and precision. Another joint fitting model technique, based on saturation and T_2_ preparation pulses, has been proposed [8] to achieve higher accuracy in the T_1_ measurement at the cost of precision (comparable to SASHA [9]).

Similarly, Magnetic Resonance Fingerprinting [10] (MRF) has also been proposed to simultaneously map multiple parameters. An MRF experiment includes three main components: 1) a variable acquisition scheme used to generate unique signal evolutions (known as *fingerprints*) for each tissue; 2) a highly undersampled acquisition trajectory that introduces spatio-temporally incoherent artefacts; and 3) a dictionary-based matching for the simultaneous estimation of multiple parameter maps. MRF with continuous data acquisition was initially proposed for simultaneous T_1_, T_2_, M_0_ and B_0_ quantification of brain imaging demonstrating reduced scan times relative to conventional methods. However, recently proposed cardiac MRF (cMRF) [[11], [12], [13]] required several adaptations to jointly map myocardial T_1_ and T_2_ relaxation times. Electrocardiogram (ECG) triggering and breath-holding are needed to minimize cardiac and respiratory motion. Variable magnetization preparation with interleaved inversion recovery (IR) and T_2_ preparation (T_2_prep) pulses are employed in cardiac MRF to increase sensitivity to T_1_ and T_2_ parameters. Furthermore, whereas dictionaries can be computed once (per sequence) ahead of time for continuous MRF, cardiac MRF requires subject-specific dictionaries that incorporate information about the heart rate variability throughout the scan.

In this study, we investigate the feasibility of a novel continuous “free-running” cardiac MRF approach (free-running cMRF) for simultaneous myocardial T_1_ and T_2_ mapping (at a given cardiac phase) and cardiac Cine imaging in a single-scan. Free-running cMRF is based on a bSSFP acquisition [14] with interrupting adiabatic IR pulses for continuous T_1_ encoding and a large range of varying flip angle for continuous T_2_ encoding. The reconstruction process uses retrospective soft-gating from a simultaneously recorded ECG signal to avoid triggering during acquisition, and a recently introduced multi-contrast patch-based undersampled reconstruction (HD-PROST [15]) combined with the MRF low rank inversion reconstruction [16,17]. Reconstructed images are then matched to a pre-computed dictionary to obtain the desired T_1_ and T_2_ estimates for a given cardiac phase. The proposed approach was tested in a standardized phantom and 10 healthy subjects, and compared to current clinical standard mapping methods.

## 2 Methods

### 2.1 Acquisition

**Fig. 1:**
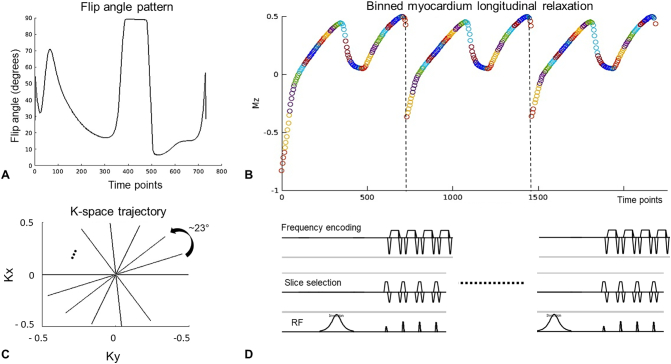
Free-running cardiac MRF acquisition scheme. A free-running acquisition is performed with an inversion pulse at the beginning of the acquisition and following inversion pulses applied every 2.9 s throughout the scan. The same flip angle train (A) is applied after each inversion pulse. In B) the longitudinal magnetization evolution of a reference myocardium tissue for the first 3 repetitions is shown (different colours represent signal corresponding to different cardiac phases). The signal is continuously sampled with a tiny golden radial (~23°) trajectory (C). The pulse diagram (D) shows the initialisation with an adiabatic hypersecant inversion pulse, and the first four RF pulses from A) showing flip angles θ(1) = θ(2)/2 and readouts balanced along all directions.

The proposed acquisition scheme is depicted in Fig. 1. Free-running cMRF consists of a continuous radial sampling of the k-space with a tiny golden angle increment of 23.63° [18] and bSSFP readouts. The flip angle pattern follows that proposed in Assländer et al. [19], although here the pattern was truncated to obtain a significant inversion of the myocardium's longitudinal magnetization at every IR pulse. Several repetitions of the same flip angle pattern (730 timepoints) are performed, each preceded by an inversion of the magnetization.

A correction for incoherent phases at the center of k-space (k = 0) [20] is performed prior to reconstruction to correct for phase and trajectory errors due to gradient delays and eddy currents. In the context of single contrast imaging an object will have a fixed phase and magnitude. In free-running cMRF experiments, phase variations can be due to varying contrasts and trajectory errors. Assuming smooth contrast variations, the original phase is shifted to a sliding window average phase. At each inversion pulse abrupt contrast and phase changes occur, therefore the sliding window average is applied for each repetition of the flip angle pattern separately (Supporting Information Fig. S1).

### 2.2 Cardiac gating

The continuously acquired data is retrospectively assigned to different cardiac phases using a simultaneously acquired ECG signal. Soft-gating [21] is performed by weighting the data acquired at time-point *t* to be assigned to the cardiac phase *p* with the weights **w**_*p*_, given by:

(1) $$w_{p}\left(t\right)=\left\{\begin{array}{cc} 1 & \mathrm{if}\;\left|t-t_{p}\right|<\frac{L_{p}}{2}\\ \begin{array}{c} \;\\ e^{-\alpha \times {\mathrm{dist}}_{p}\left(t\right)}\; \end{array} & \;\mathrm{if}\;e^{-\alpha \times {\mathrm{dist}}_{p}\left(t\right)}>\tau \;\mathrm{and}\;\left|t-t_{p}\right|>\frac{L_{p}}{2}\\ 0 & \mathrm{otherwise} \end{array}\right)$$

where *α* is a scaling factor, τ a threshold value to discard low weighted time-points, *L*_*p*_ the minimum acquisition window of cardiac phase *p*, *dist*_*p*_ the normalised distance between time-point *t* and cardiac phase *p*, and *t*_*p*_ the center of cardiac phase *p*.

### 2.3 Reconstruction

MRF time-series images for a given cardiac phase are reconstructed using low-rank MRF inversion [17] and a recently introduced multi-contrast patch-based undersampled reconstruction [15].

#### 2.3.1 Low rank inversion reconstruction

Let **x** ′ ∈ *ℂ*^*N*_*x*_*N*_*y*_*N*_*t*_^ be the N_t_ 2D time-point MRF images (vectorized), of dimensions N_x_ and N_y_, to be reconstructed. MRF time-series images are usually highly correlated. This has been demonstrated using a singular value decomposition (SVD) along the temporal dimension of the MRF dictionary **D** = ***USV***^***H***^ [16]. The low rank inversion (LRI) [17] method takes advantage of this observation to reconstruct R (with R ≪ N_t_) singular images defined as $x={U_{R}}^{H}x\prime$ (instead of the entire MRF time-series) where ***U***_*R*_ ∈ *ℂ*^*N*_*i*_*N*_*t*_×*N*_*i*_*R*^ is the block matrix applying the left singular vector matrix ***U*** truncated to an appropriate rank R to all pixels of the image series **x**′, with *N*_*i*_ = *N*_*x*_*N*_*y*_*N*_*c*_ the multi-coil image size and *N*_*c*_ the number of coils. The LRI reconstruction problem is formulated as:

(2) $$\hat{x}={\mathrm{argmin}}_{x}\;\frac{1}{2}{\left\|AU_{R}\mathrm{FCx}-k\right\|}_{2}^{2}$$

where ***k***∈ *ℂ*^*N*_*k*_*N*_*t*_^ is the undersampled k-space data, ***C***∈ *ℂ*^*N*_*i*_*R*×*N*_*x*_*N*_*y*_*R*^ the coil sensitivity maps, ***F*** ∈ *ℂ*^*N*_*i*_*R*×*N*_*i*_*R*^ the Fourier transform and ***A*** ∈ *ℂ*^*N*_*k*_*N*_*t*_×*N*_*i*_*N*_*t*_^ is the sampling operator, *N*_*k*_ = *N*_*f*_*N*_*p*_*N*_*c*_ the multi-coil k-space size, and *N*_*f*_ and *N*_*p*_ being respectively the number of samples along the frequency and phase encoding directions.

In free-running cMRF a specific dictionary **D**_*p*_ for a given cardiac phase *p* is obtained by applying the soft-gating **w**_*p*_ to each fingerprint of the dictionary **D** along the time dimension. ***U***_*Rp*_ is thus obtained from the SVD of **D**_*p*_ and the LRI reconstruction of ***x***_*p*_ the singular images for a given cardiac phase *p* is given by:

(3) $$\hat{x_{p}}={\mathrm{argmin}}_{x_{p}}\;\frac{1}{2}{\left\|W_{p}\left(AU_{\mathrm{Rp}}\mathrm{FC}x_{p}-k\right)\right\|}_{2}^{2}$$

Here **W**_*p*_ ∈ *ℂ*^*N*_*k*_*N*_*t*_×*N*_*k*_*N*_*t*_^ is the diagonal matrix applying the soft-gating vector **w**_*p*_ to all k-space points.

#### 2.3.2 Multi-contrast patch-based reconstruction: HD-PROST

**Fig. 2:**
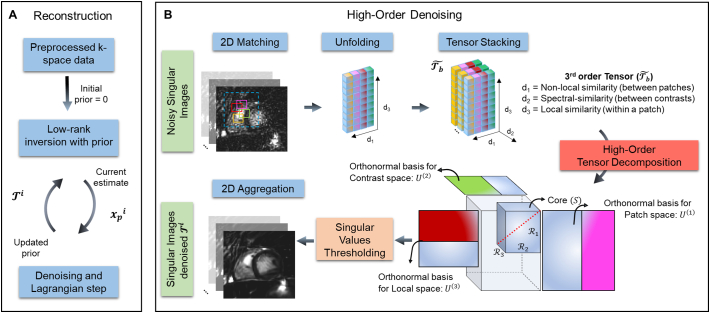
A) Reconstruction scheme for free-running cardiac MRF with two alternating optimizations. Optimization 1: Low rank inversion with prior-based regularization for reconstruction of compressed singular images ***x***_***p***_ for each cardiac phase p. Optimization 2 (B): High-order denoising/de-aliasing, where a three-dimensional tensor is created by stacking similar patches within a search window (dashed blue box) for multiple contrasts. A high order low rank assumption is made along 3 dimensions: locally (within a patch), non-locally (between similar patches in a neighbourhood) and along different contrasts of the singular images at iteration i (***x***_***p***_^i^). A low-rank tensor, for each patch, is obtained by singular value thresholding. At each iteration the denoised/de-aliased images $T^{i}$ are obtained by re-aggregating the denoised/de-aliased patches and is then used as prior for the low rank inversion reconstruction in Optimization 1. (For interpretation of the references to colour in this figure legend, the reader is referred to the web version of this article.)

To further improve the reconstruction, a high order patch-based regularization (Fig. 2) was incorporated into the LRI formulation for each cardiac phase [15]. HD-PROST iterates between a 1) multi-contrast regularized LRI reconstruction and 2) a multi-contrast tensor-based denoising/de-aliasing step that is used as prior to regularise the optimization problem in step 1. This denoising step exploits local (within a patch), non-local (between similar patches within a neighbourhood) [22,23] and contrast (between different singular images) [[24], [25], [26]] related redundancies for denoising/de-aliasing through a higher order singular value decomposition. Briefly, HD-PROST reconstruction for a given cardiac phase is formulated as:

(4) $$\begin{array}{l} L\left({\hat{x}}_{p},{\hat{T}}_{b}\right)≔\underset{x_{p},T_{b}}{\text{argmin}}\;\frac{1}{2}{\left\|W_{p}\left(AU_{\mathrm{Rp}}\mathrm{FC}x_{p}-k\right)\right\|}_{2}^{2}+\lambda \sum_{b}{\left\|T_{b}\right\|}_{*}\\ s.t.T_{b}=P_{b}\left(x_{p}\right) \end{array}$$

where the operator ***P***_*b*_(.) constructs a third order tensor $T_{b}$ composed of similar multi-contrast patches from the patch centred on voxel *b* and λ is the corresponding regularization parameter promoting low-rank regularization. More details about the implementation of HD-PROST can be found in Bustin et al. [15].

### 2.4 MRF parameter mapping reconstruction

#### 2.4.1 Signal model

The MRF signal evolution was simulated using a Hybrid-State Free Precession (HSFP) framework [14], assuming a simplified single compartment model and adiabatic transitions between steady states. An analytical solution to the transient behaviour of the magnetization vector **r** in the spherical coordinates (**e**_r_, **e**_ϑ_, **e**_ϕ_) is given by:

(5) $$r\left(t\right)=a\left(t\right)\left(r\left(0\right)+\frac{1}{T_{1}}\int_{0}^{t}\frac{\cos\vartheta \left(\tau \right)}{a\left(\tau \right)}\;\mathrm{d\tau}\right)$$

and

(6) $$a\left(\rho \right)=\exp\left(-\int_{0}^{\rho }\;\frac{\sin^{2}\vartheta \left(\varepsilon \right)}{T_{2}}+\frac{\cos^{2}\vartheta \left(\varepsilon \right)}{T_{1}}\;\mathrm{d\varepsilon}\right)$$

where *t* is the time elapsed since the last inversion pulse, *r* is the magnitude of the magnetization vector **r** along **e**_r_, *r*(0) is the magnitude after the last inversion pulse and *ϑ* is its polar angle with respect to the orientation of the main magnetic field. In HSFP, *ϑ*(*t*) is determined by the flip angle θ(t) and is set as $\vartheta \left(t\right)=\frac{\theta \left(t\right)}{2}$ due to the 180° phase cycling. Assuming on-resonance spins the evolution of the signal *s* is given by *s*(*t*) = *r*(*t*) sin *ϑ*(*t*).

Adiabatic hyperbolic hypersecant inversion pulses are employed in the proposed free-running cMRF. To correctly describe the signal evolution of low T_1_ and T_2_ components, a Bloch simulation of this pulse is considered. The inversion efficiency of the pulse obtained from simulation and defined as $\delta \left(T_{1},T_{2}\right)=\frac{r\left(t+,T_{1},T_{2}\right)}{r\left(t-,T_{1},T_{2}\right)}$ where t- and t+ are the moments right before and after the inversion pulse, is then incorporated in the HSFP simulations by replacing *r*(0) accordingly after each inversion. A slice profile correction for accurate representation and matching of the signal [27] was also incorporated by simulating for each RF pulse, N_i_ isochromats (N_i_ = 51 here) equally spaced along the slice selection axis.

#### 2.4.2 T_1_/T_2_ maps generation

Matching between each pixel of the reconstructed singular images ${\hat{x}}_{p}$ and the temporally compressed dictionary $d_{p}={U_{\mathrm{Rp}}}^{H}D_{p}$ is performed to generate the maps for a given cardiac phase *p*. Compressed time-series and dictionary entries are all normalised and an exhaustive search of ***d***_*p*_ is performed to obtain the parameter values corresponding to the maximum of the magnitude value of the complex dot product.

### 2.5 Experiments

Phantom and in-vivo acquisitions in 10 healthy subjects (30 ± 3 years, 4 males) were performed on a 1.5 T MR scanner (Ingenia, Philips Healthcare, Netherlands). Data were acquired with 28-channel cardiac and posterior coils. Written informed consent was obtained from all participants before undergoing the MR scans and the study was approved by the Institutional Review Board.

#### 2.5.1 Phantom acquisition

Experiments were performed in a T_1_/T_2_ standardized (T1MES) phantom [28]. This phantom includes T_1_ and T_2_ values typical of cardiac acquisitions such as blood (T_1_ = 1489 ms, T_2_ = 243 ms) and myocardium (T_1_ = 1090 ms, T_2_ = 48 ms). Free-running cMRF, conventional 2D ECG-triggered MOLLI [5(3)3] [6] and SASHA [9] T_1_ maps, as well as T2-GRASE [29] maps were acquired with the same resolution (2 × 2 × 10 mm^3^) for comparison purposes. Sequences were repeated three times to assess repeatability of the measurements in phantom.

Free-running cMRF imaging parameters were set as followed: spatial resolution 2 × 2 mm^2^, slice thickness 10 mm, field of view (FOV) = 288 × 288 mm^2^, bandwidth (BW) = 542.5 Hz/pixel, TR = 4 ms, echo time (TE) = 1.99 ms, acquisition time 29.4 s. Inversion recovery spin echo measurements for reference T_1_ values and spin echo for T_2_ values are provided by the manufacturer [28]. T_1_ spin echo (IRSE) acquisition parameters include: TR = 10s, TE = 14.75 ms, 8 inversion delays in the range of 25 ms to 3200 ms. T_2_ spin echo (SE) acquisition parameters include 8 echo times ranging from 10 to 640 ms. Acquisition parameters for MOLLI included: TR/TE = 2.4/1.19 ms, flip angle (FA) = 35°, SENSE factor = 2, BW = 1085 Hz/pixel. Acquisitions parameters for SASHA included: TR/TE = 2.4/1.19 ms, saturation times: 120:60:650 ms (last saturation times only when applicable due to heartrate constraints) and infinity image, FA = 70°, SENSE factor = 2, BW = 1085 Hz/pixel. Acquisitions parameters for T2-GRASE included: TEs = 8.3:8.3:74.7 ms, EPI factor = 7, FA = 90°, SENSE factor = 2.4 and double inversion recovery for blood signal nulling.

#### 2.5.2 In vivo acquisitions

In vivo scans were performed in short-axis orientation. Free-running cMRF acquisition was performed after hyperventilation [30]. Acquisition parameters for free-running cMRF were set as for the phantom experiment. Conventional MOLLI [5(3)3] and SASHA T_1_ maps, as well as T_2_-GRASE maps were acquired in diastole for all subjects. A prospectively gated 2D bSSFP Cine scan was obtained for comparison purposes in eight out of ten subjects. Cine acquisition parameters included: TR/TE = 2.8/1.39 ms, FA = 60°, SENSE factor = 2, BW = 1377 Hz/pixel, eight cardiac phases.

#### 2.5.3 Free-running T_1_ and T_2_ cMRF reconstruction

The MRF dictionary **D** was computed once for a range of T_1_s of [50:10:1400, 1430:30:1600, 1700:100:2200, 2400:200:3000] ms and a range of T_2_s of [5:2:80, 85:5:150, 160:10:300, 330:30:600] ms. The specific T_1_ and T_2_ values of the standardized phantom were also included in the dictionary.

Free-running cMRF T_1_ and T_2_ maps were reconstructed at diastole using the recorded or simulated (phantom) ECG signal and by selecting cardiac phase *p* = 6 of 8 with *L*_*p*_=1/8th of the cardiac cycle. The simulated ECG signal for the phantom experiments corresponded to a fixed heartrate of 65 beats per minutes. Reconstruction parameters were set empirically in one data set and used for the rest of the acquisitions. The rank R was set to 6 and the soft-gating parameters α and τ were set to 4.5 and 0.5 respectively, resulting in a soft weighted acquisition window of 27% of the cardiac cycle (with 12.5% fully weighted). HD-PROST reconstruction parameters were set empirically following those employed in Bustin et al. [15], i.e. λ = 5e^−3^, patch size = 5 × 5, search window = 20 × 20; number of selected similar patches = 20, ADMM iterations = 6. Reconstruction was performed using MATLAB (The MathWorks Inc) and took ~22 min per cardiac phase, including 21 min for HD-PROST reconstruction and 1.7 s for matching on a Linux workstation with 8 Intel Xeon E5-2687W (3.1 GHz) and 252 GB RAM.

#### 2.5.4 Free-running Cine cMRF reconstruction

A bSSFP contrast was synthetically generated from T_1_, T_2_ and M_0_ maps obtained from free-running cMRF using the bSSFP analytical on-resonance signal equation [31]:

(8) $$S_{\mathrm{bSSFP}}=M_{0}\frac{\sqrt{E_{2}\left(1-E_{1}\right)\text{sinθ}}}{1-E_{1}E_{2}\left(E_{1}-E_{2}\right)\cos\theta \;}$$

where *E*_1, 2_ = *e*^−*TR*/*T*_1, 2_^ and θ is the nominal flip angle.

The synthetic bSSFP was generated using TR = 2.5 ms and θ=60° and 8 cardiac phases.

Other synthetically generated contrasts (e.g. black-myocardium and black-blood) can also be generated from T_1_, T_2_ and M_0_ maps obtained with the proposed free-running cMRF, as described in Supporting Information Text S1.

#### 2.5.5 Analysis

Regions of interest (ROIs) were drawn in the 9 phantom vials and in the septum of 10 healthy subjects. Free-running cMRF T_1_ and T_2_ measurements were compared to conventional measurements through coefficients of determination (r^2^), intra-class correlation coefficients (ICC), Bland-Altman plots, boxplots, mean bias and standard deviation (SD) of the measurements. The ICC(A, 1) was used when comparing results to the trusted reference phantom spin echo measurements, and the ICC(A, k) when assessing repeatability (as defined in [32]). An estimated 2D ejection fraction (EF) is computed from the diastolic and systolic areas obtained from the synthetic bSFFP and conventional Cine scans. A Bland-Altman plot, coefficient of determination and ICC(A, k) are reported. Paired *t*-tests comparing T_1_, T_2_ and 2D EF measurements were performed to assess if the results are statistically significant (*p* < .05; or *p* < .025 for T_1_ comparisons using the Bonferroni correction).

## 3 Results

### 3.1 Phantom study

**Fig. 3:**
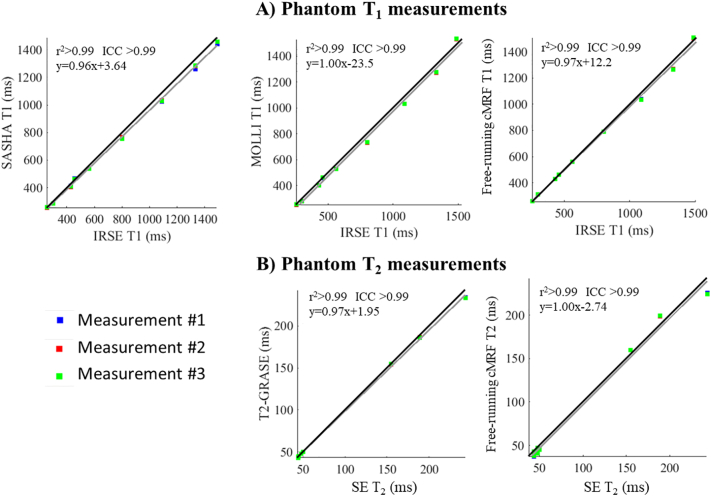
Correlation plots for A) T_1_ and B) T_2_ measurements showing 3 repeated measurements with the proposed approach, MOLLI, SASHA and T2-GRASE compared to reference spin echo measurements. The three repeated measurements for the proposed free-running cMRF and the conventional methods are in good agreement with the reference measurements (r^2^ > 0.99 and ICC(A,1) > 0.99). Repeatability (using the three repeated measurements) for each method is assessed in Supporting Information Fig. S2.

T_1_ and T_2_ measurements of the standardized phantom with the proposed and conventional approaches in comparison to the gold standard spin echo measurements are shown in Fig. 3. Correlation analysis (r^2^ > 0.99, ICC(A,1) > 0.99 for T_1_ and T_2_) indicated good agreement of the proposed and conventional methods with the reference methods. Accurate T_1_ and T_2_ values were obtained for all but the highest T_2_ values. When considering all vials small biases (−11 ms for T_1_ and − 3.1 ms for T_2_) were observed. Bland Altman plots (Supporting Information Fig. S2) comparing the three free-running cMRF measurements showed very high correlation (ICC(A,k) > 0.99) and low 1.96SD of 1.9 ms for T_1_ and 0.57 ms for T_2_, indicating similar repeatability to MOLLI (1.96SD = 2.2 ms) and T2-GRASE (1.96SD = 0.37 ms) in phantom.

### 3.2 In vivo study

**Fig. 4:**
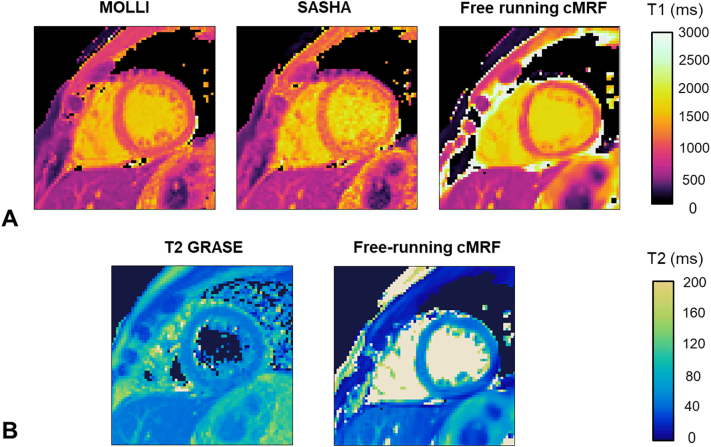
A) From left to right T_1_ maps obtained with: MOLLI, SASHA and the proposed free-running cardiac MRF. B) From left to right T_2_ maps obtained with: T2-GRASE and the proposed free-running cMRF.

**Fig. 5:**
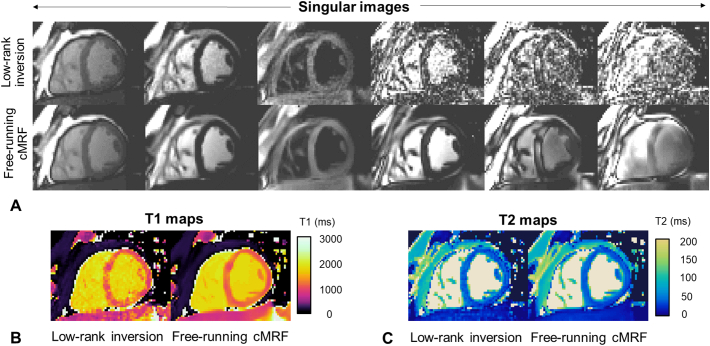
A) Singular images (1 to 6, from left to right) reconstructed with low-rank inversion (LRI) reconstruction and the proposed free-running cardiac MRF (cMRF) approach. Increased sharpness and strong denoising of singular images, especially at a rank higher than 2 can be observed for the reconstruction used in the proposed free-running cMRF framework. B) and C) show the corresponding T_1_ and T_2_ maps.

T_1_ and T_2_ maps obtained with the proposed free-running cMRF are compared to MOLLI, SASHA and T2-GRASE maps for a healthy subject in Fig. 4. Singular value images and corresponding T_1_ and T_2_ maps from LRI and HD-PROST reconstruction are shown in Fig. 5 for a different subject.

**Fig. 6:**
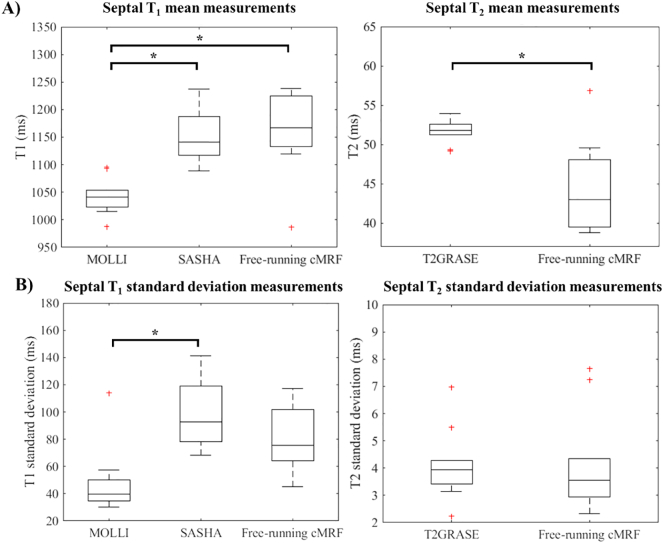
Boxplots showing septal T1 and T2 mean (A) and standard deviation (B) obtained in 10 healthy subjects using the proposed free-running cMRF and the conventional methods. In each box plot, the horizontal line depicts the median, the top and bottom of the box represent the upper and lower quartiles, whereas the whiskers indicate the minimum and maximum values excluding outliers. Additional outliers are indicated by (+). Statistically significant differences (paired *t*-test) are indicated with * (*p* < .025 for T_1_ and *p* < .05 for T_2_ comparisons).

Over all subjects, T_1_ average measurements and mean SD values were 1043 ± 48 ms, 1150 ± 100 ms and 1160 ± 79 ms for MOLLI, SASHA and free-running cMRF respectively with no significant differences between SASHA and free-running cMRF (*p* = .7326). T_2_ values were 51.7 ± 4.1 ms and 44.6 ± 4.1 ms for T_2_-GRASE and free-running cMRF respectively. The negative bias observed in T_2_ (−7.1 ms) was statistically significant (*p* = 4.7E^−3^). Tukey boxplots (Fig. 6) summarizes the in-vivo T_1_ and T_2_ mean and standard deviation measurements for the different methods and statistically significant differences.

**Fig. 7:**
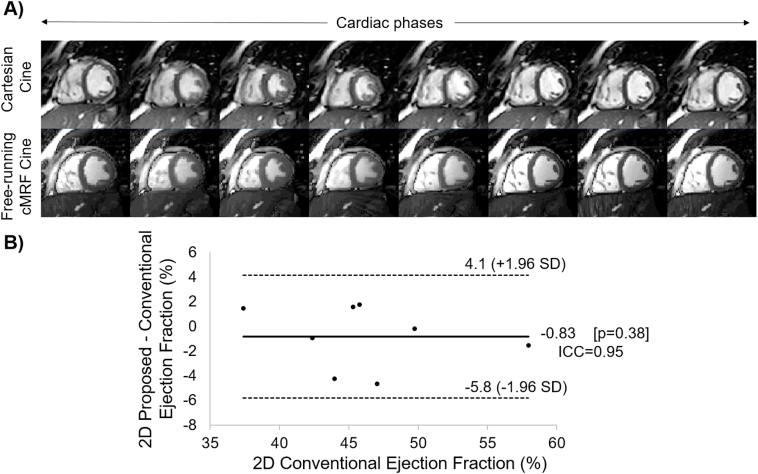
A) Conventional Cartesian Cine scan in comparison to the proposed free-running cardiac MRF Cine sequence for 8 cardiac phases. B) Bland Altman plot comparing the proposed and conventional 2D ejection fraction measurements in 8 subjects. High correlation (ICC(A, k) = 0.95) and no statistical differences (*p* = .38) were observed between methods.

Free-running cMRF Cine images are compared to conventional Cartesian Cine imaging in Fig. 7.A. The resulting measured 2D EF was compared using a Bland-Altman plot (Fig. 7.B) showing good agreement (ICC(A, k) = 0.95, mean bias = −0.83%) between the two methods with no statistically significant differences (*p* = .38).

**Fig. 8:**
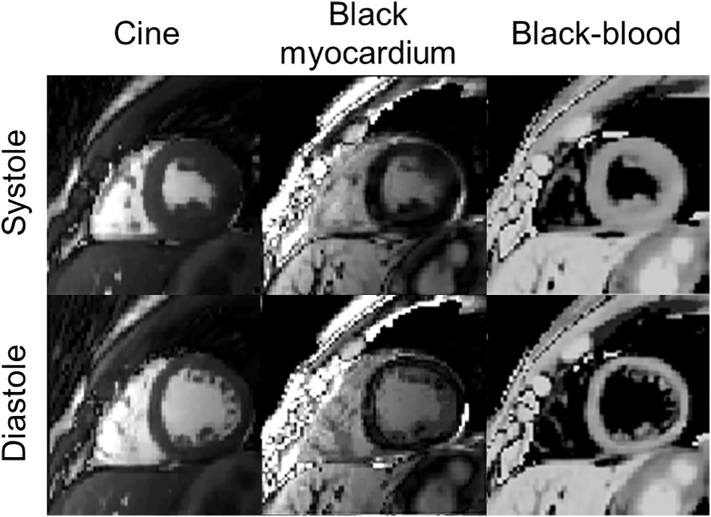
Systolic (top) and diastolic (bottom) synthetic contrasts generated from the proposed free-running cardiac MRF acquisition. From left to right: Cine (bSSFP with 60° flip angle), black myocardium and black blood images (see Supporting Information Text S1).

Black-myocardium and black-blood synthetic contrast images generated from the proposed free-running cMRF acquisition are shown at systole and diastole in Fig. 7 and for eight cardiac phases in Supporting Information Fig. S1. Corresponding free-running cMRF Cine images are also included in Fig. 8 and Supporting Information Fig. S3.

T_1_ and T_2_ maps are generated at diastole with the proposed approach, however there is the potential to generate maps at different cardiac phases as shown in Supporting Information Figure. S4 (for the same subject as in Supporting Information Figure. S3) and Supporting Information Supporting Information Video S1, Supporting Information Video S2 for two different subjects.

## 4 Discussion

This study presents a novel non-ECG triggered 2D magnetic resonance fingerprinting (MRF) sequence for simultaneous and inherently co-registered myocardial T_1_ and T_2_ mapping and cardiac Cine imaging. Phantom results indicate good agreement with the gold standard sequences for both T_1_ and T_2_ measurements with accuracy and precision comparable to clinically used methods.

While rewound gradient echo was used in previously proposed ECG-triggered cardiac MRF [11], bSSFP was employed in this study. Balanced acquisitions in the context of MRF presents higher resilience to through plane motion [33] and are intrinsically flow corrected. Balanced sequences also have less diffusion weighting [34], and higher SNR. However, they are more sensitive to B_0_ and B_1_ inhomogeneities. A short, fixed TR of 4 ms with smooth flip angle variations was used to mitigate sensitivity to B_0._ However, corrections for B_0_ and B_1_ inhomogeneities were not considered in this study and are possible sources of bias.

In vivo myocardial septal measurements showed no statistically significant differences between free-running cMRF and SASHA (*p* = .7326) mean T_1_ and between free-running cMRF and conventional Cine (*p* = .38) 2D EF measurements (Fig. 6). A statistically significant bias compared to T2-GRASE (−7.1 ms (*p* = 4.7E^−3^)) is reported in this study. Similar biases in T_2_ have been also reported in previous MRF [35,36] and cardiac MRF studies [11,37,38]. In-vivo precision was assessed through standard deviation measured in the septum, which is a suboptimal but accessible surrogate for precision, and indicated lower precision of free-running cMRF in vivo (79 ms for T_1_ and 4.1 ms for T_2_) compared to the values obtained in phantom experiments (1.9 ms for T_1_ and 0.56 ms for T_2_). These larger biases and lower precision observed in vivo compared to phantom results can be attributed to confounding factors such as partial volume effects [39], higher field inhomogeneities, magnetization transfer [40,41], diffusion [34], flow [42] and to remaining in plane and through plane motion during the scan [33,43]. Through plane motion in 2D MRF brings fresh magnetization into the excited slice which will corrupt the acquired MRF signal irreversibly and bias the estimated parameter maps [33,43]. Blood entering and leaving the imaging slice (potentially crossing the acquisition plane multiple times during a heartbeat) could not be modelled (similar to previous cMRF approaches [11,37]) and larger biases were observed in the blood pools than in the myocardium. In this study, we chose a large slice thickness (similar to conventional triggered cardiac T_1_ and T_2_ mapping) to mitigate the bias induced by through-plane motion on the myocardium measurements, however this will always affect 2D scans. To avoid biases due to cardiac motion across the cardiac cycle, free-running 3D MRF approaches will be investigated in future works. The results of this study show the feasibility of 2D free-running cardiac MRF, warranting future extensions to 3D.

In contrast to previously proposed cardiac MRF [11], the proposed approach does not require ECG triggering. The proposed non ECG-triggered cardiac MRF offers the opportunity of evaluating cardiac function and tissue properties simultaneously. Moreover, the proposed approach does not require a patient specific dictionary, which may become computationally demanding as more parameters are considered in the MRF signal model. Consequently, it should be simpler to simulate longer acquisitions (e.g. those needed for 3D scans) and incorporate additional MRF parameters (e.g. MT, diffusion) and corrections into a free-running cardiac MRF acquisition.

One of the limitations of this study is the low temporal resolution of the Cine images. In the proposed reconstruction, the temporal resolution is automatically adapted to the patient's heartrate and is between 12.5% and 27% of the mean cardiac cycle (i.e. between 125 ms and 270 ms for a heartrate of 60 bpm). This temporal resolution is low in comparison to conventional cardiac Cine functional imaging (usually ~50 ms) and leads to some blurring of the images however no statistically significant bias were observed in the 2D EF measurements between the proposed approach and conventional Cine imaging. Another limitation of this study is the long acquisition time. Although long breath-holds are attainable in patient populations using hyperventilation, as shown by Fischer et al. [30], reducing acquisition time and increasing temporal resolution by exploiting anatomical and relaxation information redundancies in the cardiac dimension will be investigated in future works to allow for further undersampling of a given cardiac phase. This could be achieved for example following a similar approach to the recently introduced cardiac MR multitasking [44] that exploits low rankness in both contrast and motion dimensions for reconstruction or extending HD-PROST by modifying the operator ***P***_*b*_(.) to exploit patch similarities also in the cardiac direction [45]. Moreover, motion alignment [46] or motion correction [43] could be exploited between cardiac phases to allow for a matching on the whole fingerprint. This could enable higher temporal resolution and shorter acquisition times.

In this study free-running cardiac MRF was performed without contrast injection. Post contrast injection applications could potentially offer shorter acquisition times due to shorter relaxation times, additional extra cellular volume (ECV) information and synthetic late gadolinium enhanced contrasts [47,48] different cardiac phases could be obtained. Such an acquisition providing tissue characterisation and function with whole heart coverage could greatly simplify the workflow of CMR exams [44] by providing comprehensive assessment of cardiac health in one acquisition potentially acquired twice (pre and post contrast).

## 5 Conclusion

A novel free-running cardiac MRF framework for simultaneous and inherently co-registered myocardial T_1_ and T_2_ mapping and cardiac Cine imaging was proposed. Accurate and precise T_1_ and T_2_ measurements were obtained in phantom and accurate left ventricular 2D ejection fraction was obtained in-vivo. The feasibility of two-dimensional free-running cardiac MRF was investigated, further extensions to three-dimensional free-running acquisitions and validation in patients is warranted.

The following are the supplementary data related to this article.

Supporting Information Video S1 Free-running cardiac MRF T_1_, T_2_ and Cine movie showing 8 cardiac phases dynamically for better visualisation of the cardiac motion for a healthy subject. Supporting Information Video S1

Supporting Information Video S2 Free-running cardiac MRF T_1_, T_2_ and Cine movie showing 8 cardiac phases dynamically for better visualisation of the cardiac motion for a second healthy subject. Supporting Information Video S2

The following document contains Supporting Information Text S1 and Supporting Information Figures S1 to S4. Image 1

## CRediT authorship contribution statement

**O. Jaubert:**Conceptualization, Methodology, Software, Data curation, Writing - original draft, Visualization, Investigation, Writing - review & editing.**G. Cruz:**Conceptualization, Methodology.**A. Bustin:**Conceptualization, Methodology.**T. Schneider:**Software.**P. Koken:**Software.**M. Doneva:**Software.**D. Rueckert:**Funding acquisition.**R.M. Botnar:**Funding acquisition.**C. Prieto:**Conceptualization, Methodology, Supervision, Funding acquisition, Writing - review & editing.

## References

[1] Pennell D.J., Sechtem U.P., Higgins C.B. (2004). Clinical indications for cardiovascular magnetic resonance (CMR): consensus panel report. Eur Heart J.

[2] Verhaert D., Thavendiranathan P., Giri S. (2011). Direct T2 quantification of myocardial edema in acute ischemic injury. JACC Cardiovasc Imaging.

[3] Haaf P., Garg P., Messroghli D.R., Broadbent D.A., Greenwood J.P., Plein S. (2017). Cardiac T1 mapping and Extracellular Volume (ECV) in clinical practice: a comprehensive review. J Cardiovasc Magn Reson.

[4] Park C.H., Choi E.-Y., Kwon H.M. (2013). Quantitative T2 mapping for detecting myocardial edema after reperfusion of myocardial infarction: validation and comparison with T2-weighted images. Int J Card Imaging.

[5] Santini F., Kawel-Boehm N., Greiser A., Bremerich J., Bieri O. (2015). Simultaneous T1 and T2 quantification of the myocardium using cardiac balanced-SSFP inversion recovery with interleaved sampling acquisition (CABIRIA). Magn Reson Med.

[6] Messroghli D.R., Radjenovic A., Kozerke S., Higgins D.M., Sivananthan M.U., Ridgway J.P. (2004). Modified Look-Locker inversion recovery (MOLLI) for high-resolutionT1 mapping of the heart. Magn Reson Med.

[7] Giri S., Chung Y.-C., Merchant A. (2009). T2 quantification for improved detection of myocardial edema. J Cardiovasc Magn Reson.

[8] Akçakaya M., Weingärtner S., Basha T.A., Roujol S., Bellm S., Nezafat R. (2016). Joint myocardial T1 and T2 mapping using a combination of saturation recovery and T2 -preparation. Magn Reson Med.

[9] Chow K., Flewitt J.A., Green J.D., Pagano J.J., Friedrich M.G., Thompson R.B. (2014). Saturation recovery single-shot acquisition (SASHA) for myocardial *T*_1_ mapping. Magn Reson Med.

[10] Ma D., Gulani V., Seiberlich N. (2013). Magnetic resonance fingerprinting. Nature.

[11] Hamilton J.I., Jiang Y., Chen Y. (2017). MR fingerprinting for rapid quantification of myocardial T1, T2, and proton spin density. Magn Reson Med.

[12] Hamilton J.I., Jiang Y., Ma D. (2018). Investigating and reducing the effects of confounding factors for robust T1 and T2 mapping with cardiac MR fingerprinting. Magn Reson Imaging.

[13] Liu Y., Hamilton J., Rajagopalan S., Seiberlich N. (2018). Cardiac magnetic resonance fingerprinting: technical overview and initial results. JACC Cardiovasc Imaging.

[14] Assländer J., Novikov D.S., Lattanzi R., Sodickson D.K., Cloos M.A. (2019). Hybrid-state free precession in nuclear magnetic resonance. Commun Phys.

[15] Bustin A., Cruz G., Jaubert O., Karina L., Botnar R.M., Prieto C. (2019). High-Dimensionality Undersampled Patch-Based Reconstruction (HD-PROST) for accelerated multi-contrast magnetic resonance imaging. Magn Reson Med.

[16] McGivney D.F., Pierre E., Ma D. (2014). SVD compression for magnetic resonance fingerprinting in the time domain. IEEE Trans Med Imaging.

[17] Assländer J., Cloos M.A., Knoll F., Sodickson D.K., Hennig J., Lattanzi R. (2018). Low rank alternating direction method of multipliers reconstruction for MR fingerprinting. Magn Reson Med.

[18] Wundrak S., Paul J., Ulrici J., Hell E., Rasche V. (2015). A small surrogate for the golden angle in time-resolved radial MRI based on generalized Fibonacci sequences. IEEE Trans Med Imaging.

[19] Assländer J., Lattanzi R., Sodickson D.K., Martijn A. (2017). Relaxation in spherical coordinates: analysis and optimization of pseudo-SSFP based MR-fingerprinting.

[20] Winkelmann S., Schaeffter T., Koehler T., Eggers H., Doessel O. (2007). An optimal radial profile order based on the Golden ratio for time-resolved MRI. IEEE Trans Med Imaging.

[21] Johnson K.M., Block W.F., Reeder S.B., Samsonov A. (2012). Improved least squares MR image reconstruction using estimates of k-Space data consistency. Magn Reson Med.

[22] Akçakaya M., Basha T.A., Goddu B. (2011). Low-dimensional-structure self-learning and thresholding: regularization beyond compressed sensing for MRI reconstruction. Magn Reson Med.

[23] Bustin A., Ginami G., Cruz G. (2019). Five-minute whole-heart coronary MRA with sub-millimeter isotropic resolution, 100% respiratory scan efficiency, and 3D-PROST reconstruction. Magn Reson Med.

[24] Tamir J.I., Uecker M., Chen W. (2017). *T*_2_ shuffling: sharp, multicontrast, volumetric fast spin-echo imaging. Magn Reson Med.

[25] Lima da Cruz G., Bustin A., Jaubert O., Schneider T., Botnar R.M., Prieto C. (2019). Sparsity and locally low rank regularization for MR fingerprinting. Magn Reson Med.

[26] Zhang T., Pauly J.M., Levesque I.R. (2015). Accelerating parameter mapping with a locally low rank constraint. Magn Reson Med.

[27] Ma D., Coppo S., Chen Y. (2017). Slice profile and B _1_ corrections in 2D magnetic resonance fingerprinting. Magn Reson Med.

[28] Captur G., Gatehouse P., Kellman P. (2016). A T1 and ECV phantom for global T1 mapping quality assurance: the T1 mapping and ECV standardisation in CMR (T1MES) program. J Cardiovasc Magn Reson.

[29] Sprinkart A.M., Luetkens J.A., Träber F. (2015). Gradient Spin Echo (GraSE) imaging for fast myocardial T2 mapping. J Cardiovasc Magn Reson.

[30] Fischer K., Yamaji K., Luescher S. (2018). Feasibility of cardiovascular magnetic resonance to detect oxygenation deficits in patients with multi-vessel coronary artery disease triggered by breathing maneuvers. J Cardiovasc Magn Reson.

[31] Scheffler K., Lehnhardt S. (2003). Principles and applications of balanced SSFP techniques. Eur Radiol.

[32] McGraw K.O., Wong S.P. (1996). Forming inferences about some intraclass correlation coefficients. Psychol Methods.

[33] Yu Z., Zhao T., Assländer J., Lattanzi R., Sodickson D.K., Cloos M.A. (2018). Exploring the sensitivity of magnetic resonance fingerprinting to motion. Magn Reson Imaging.

[34] Kobayashi Y., Terada Y. (2019). Diffusion-weighting caused by spoiler gradients in the fast imaging with steady-state precession sequence may lead to inaccurate T2 measurements in MR fingerprinting. Magn Reson Med Sci.

[35] Chen Y., Jiang Y., Pahwa S. (2016). MR fingerprinting for rapid quantitative abdominal imaging. Radiology.

[36] Körzdörfer G., Kirsch R., Liu K. (2019). Reproducibility and repeatability of MR fingerprinting relaxometry in the human brain. Radiology.

[37] Jaubert O., Cruz G., Bustin A. (2019). Water–fat Dixon cardiac magnetic resonance fingerprinting. Magnetic resonance in medicine.

[38] Cruz G., Jaubert O., Botnar R.M., Prieto C. (2019). Cardiac magnetic resonance fingerprinting: technical developments and initial clinical validation. Curr Cardiol Rep.

[39] Tang S., Fernandez-Granda C., Lannuzel S. (2018). Multicompartment magnetic resonance fingerprinting. Inverse Prob.

[40] Malik S.J., Teixeira R.P.A.G., Hajnal J.V. (2018). Extended phase graph formalism for systems with magnetization transfer and exchange. Magn Reson Med.

[41] Hilbert T., Xia D., Block K.T. (2019). Magnetization transfer in magnetic resonance fingerprinting. Magnetic resonance in medicine.

[42] Flassbeck S., Schmidt S., Bachert P., Ladd M.E., Schmitter S. (2019). Flow MR fingerprinting. Magn Reson Med.

[43] Cruz G., Jaubert O., Schneider T., Botnar R.M., Prieto C. (2019). Rigid motion-corrected magnetic resonance fingerprinting. Magn Reson Med.

[44] Christodoulou A.G., Shaw J.L., Nguyen C. (2018). Magnetic resonance multitasking for motion-resolved quantitative cardiovascular imaging. Nat Biomed Eng.

[45] Jaubert O., Cruz G., Bustin A. (2019). Cardiac motion resolved magnetic resonance fingerprinting with joint reconstruction: jMORE-MRF. Proceedings of the 27th annual meeting of ISMRM Montreal, Canada.

[46] Becker K.M., Blaszczyk E., Funk S. (2019). Fast myocardial T _1_ mapping using cardiac motion correction. Magn Reson Med.

[47] Weingärtner S., Yaman B., Shenoy C. (2018). Cardiac phase-resolved late-gadolinium enhancement imaging. Proceedings of the 26th annual meeting of ISMRM Paris, France.

[48] Abdula G., Nickander J., Sörensson P. (2018). Synthetic late gadolinium enhancement cardiac magnetic resonance for diagnosing myocardial scar. Scand Cardiovasc J.

