# Supplementary material for: Free-running cardiac magnetic resonance fingerprinting: Joint T1/T2 map and Cine imaging
Source: Magn Reson Imaging. 2020 May;68:173–82. doi: 10.1016/j.mri.2020.02.005 (PMC7677167; doi:10.1016/j.mri.2020.02.005)
Supplement: Supplementary file 3 — The following document contains Supporting Information Text S1 and Supporting Information Figures S1 to S4. [file mmc3.docx]

# Supporting Information Figures and Videos

## Supporting Information Text S1

Synthetic contrasts, designed for specific purposes, can be generated from the T_1_, T_2_ and M_0_ maps obtained with free-running cMRF. In Supporting Information Figure S1, three synthetic contrasts are proposed: 1) The conventional Cine bSSFP contrast with FA=60^o^ and TR=2.5 ms, 2) A black-myocardium Cine image may enable scar delineation throughout the cardiac cycle (47,48) if performed after contrast agent administration (demonstrated without contrast injection in Supporting Information Figure S3), 3) and a fictive black blood contrast which uses T_1_ and T_2_ for more robust tissue differentiation and better blood nulling are shown. The proposed realistic and fictive contrasts are derived using equations S1.1 and S1.2 and are shown in Supporting Information Figure S1:

| $S_{LGE}=M_{0}\left( 1-2\exp\left( -\frac{ln(2)\times T_{1Myocardium}}{T_{1}} \right) \right)$ | [S1.1] |
| --- | --- |

| $S_{BB}=2-\left( \frac{T_{1}}{T_{1Blood}}+\frac{T_{2}}{T_{2Blood}} \right)$ | [S1.2] |
| --- | --- |

## Supporting Information Figure S1


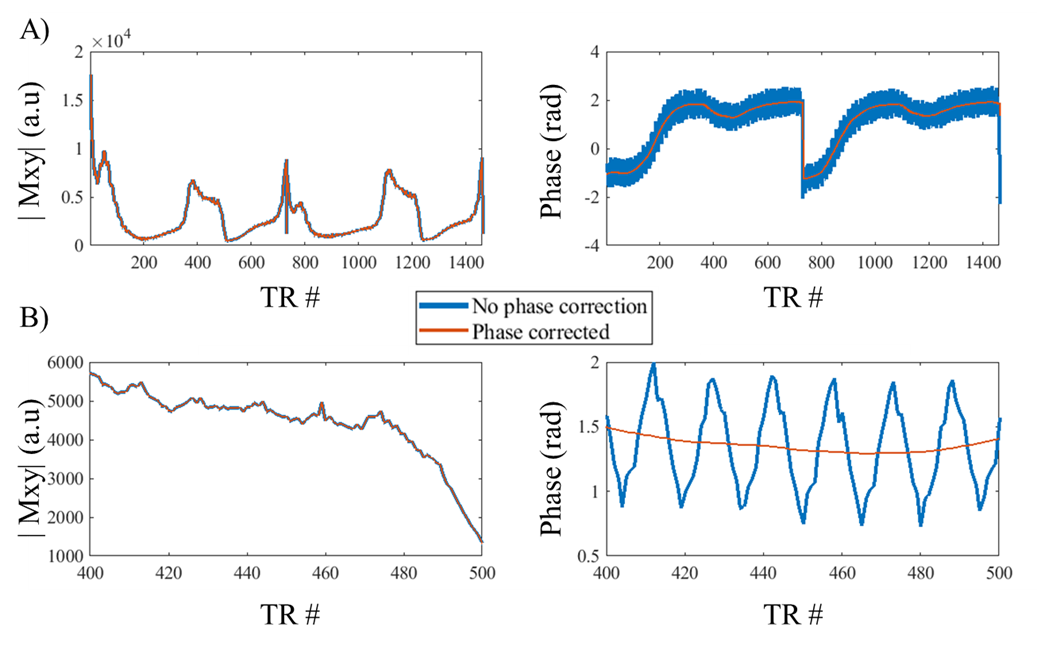


Supporting Information Figure S1. A) Magnitude and phase of the center of k-space for the first 1460 acquired radial spokes showing the effect of phase correction. Note the inversion between timepoints 730 and 731 where sliding window is not applied. B) Zoom in on timepoints 400 to 500 to visually observe the regular phase oscillations due to trajectory errors and effect of correction.

## Supporting Information Figure S2


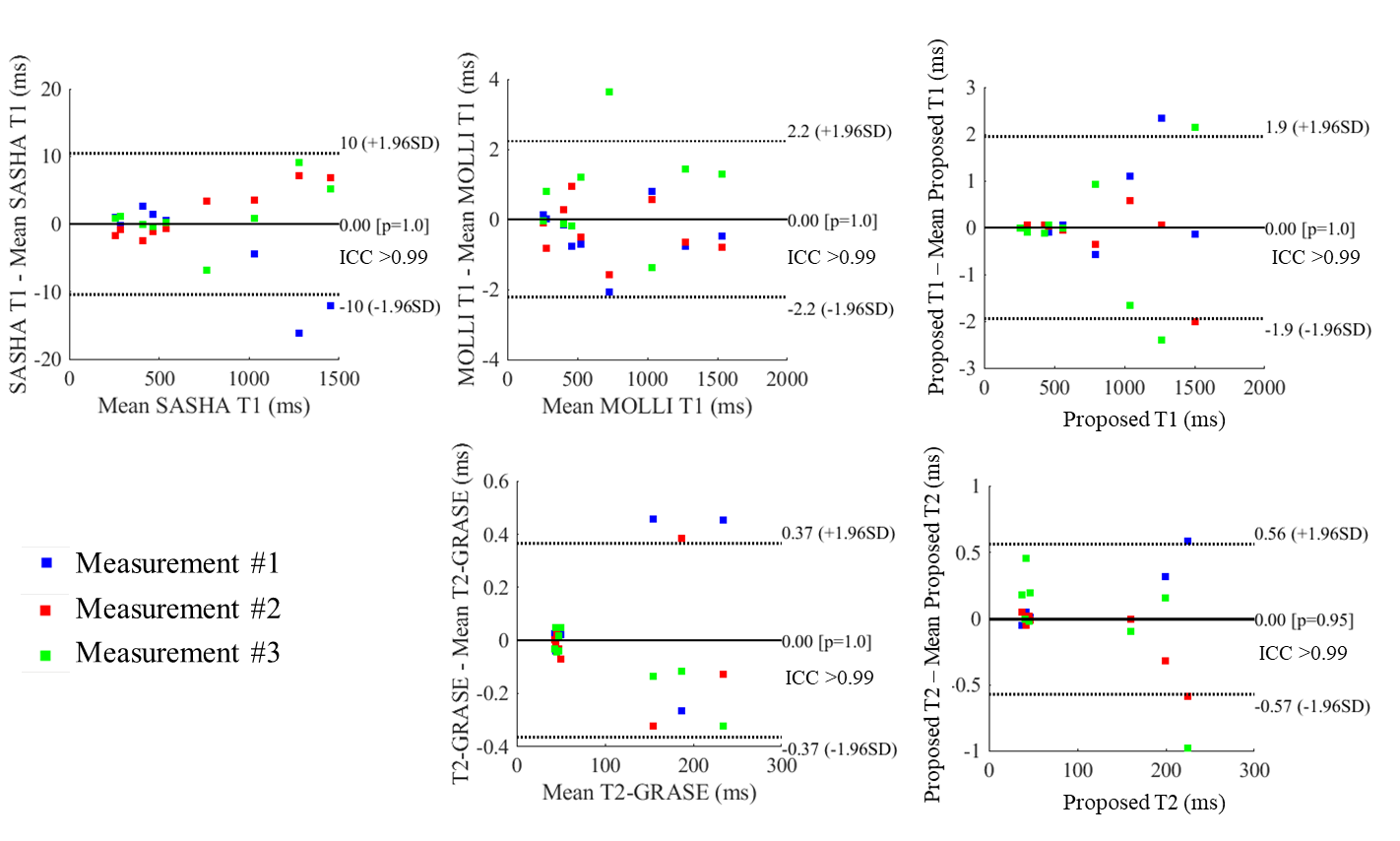


Supporting Information Figure S2. Bland Altman plots comparing the repeatability of the measurements in phantom for the different methods (compared to the mean measurement). The 1.96 standard deviation was found to be 10 ms, 2.2 ms and 1.9 ms for SASHA, MOLLI and free-running cMRF T_1_ measurements and 0.37 ms and 0.57 ms for T2-GRASE and free-running cMRF T_2_ measurements. Please note that the vertical scales change between the plots.

## Supporting Information Figure S3

**
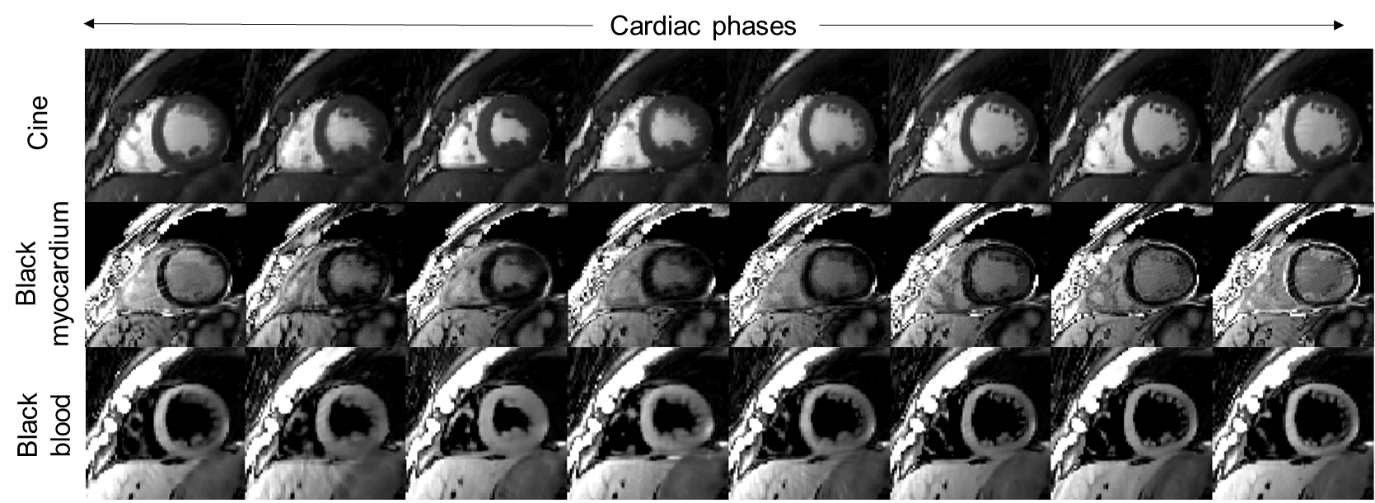
**Supporting Information Figure S3. Synthetic Cine, black-myocardium and black-blood for 8 cardiac phases generated from the proposed free-running cMRF T_1,_ T_2_ and M_0_ maps.

## Supporting Information Figure S4


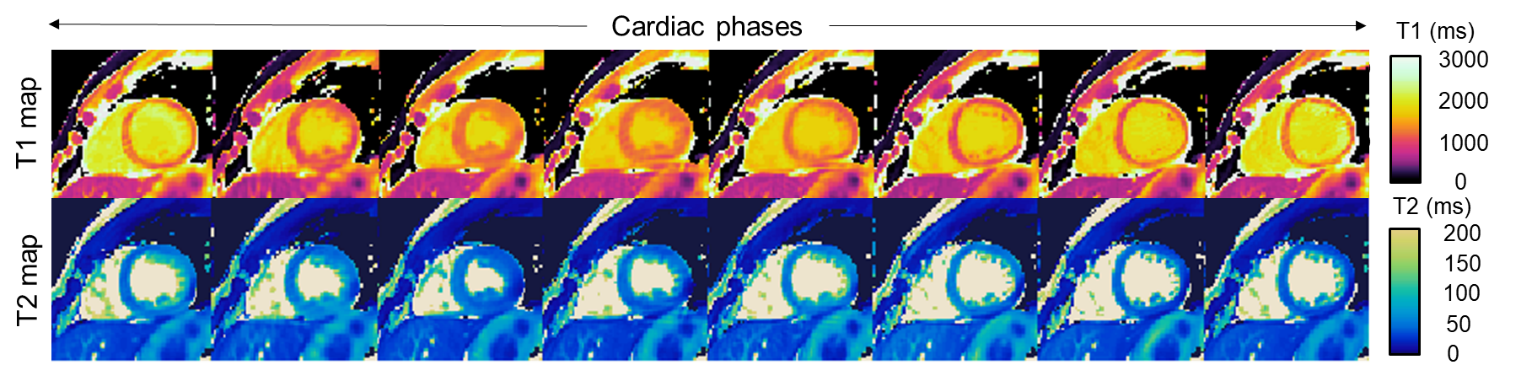


Supporting Information Figure S4. T_1_ and T_2_ maps for 8 cardiac phases generated from the proposed free-running cMRF scan.
